# Caries Experience among Children with History of Neonatal Stunting

**DOI:** 10.1055/s-0042-1750775

**Published:** 2022-09-08

**Authors:** Siska Yohana, Ratna Indriyanti, Netty Suryanti, Laili Rahayuwati, Neti Juniarti, Arlette S. Setiawan

**Affiliations:** 1 Dentist Education Program, Faculty of Dentistry, Universitas Padjadjaran, Jawa Barat, Indonesia; 2 Department of Pediatric Dentistry, Faculty of Dentistry, Universitas Padjadjaran, Jawa Barat, Indonesia; 3 Department of Community Dentistry, Faculty of Dentistry, Universitas Padjadjaran, Jawa Barat, Indonesia; 4 Department of Community Nursing, Faculty of Nursing, Universitas Padjadjaran, Jawa Barat, Indonesia

**Keywords:** neonatal stunting, malnutrition, dental caries, dental caries index

## Abstract

**Objective** Children with neonatal growth retardation (defined as birth length <48cm) are at risk for chronic malnutrition that begins before birth and continues into infancy. Stunting can adversely affect a child's growth and development, including oral health itself, and especially the experience of dental caries. This study analyzed the dental caries experience in children with neonatal growth retardation.

**Materialsand Methods** This was a baseline and 1-year follow-up analysis of a cohort of stunted children in a potential stunting site in Bandung City. Annual data collection included interviews with mothers and dental and anthropometric examinations of children. Descriptive analysis was performed in SPSS.

**Statistical Analysis** Data were recorded on paper forms and manually entered into a Microsoft Excel spreadsheet for later analysis using IBM SPSS (version 23.0). After assessment, descriptive statistics was generated prior to bivariate analysis.

**Results** Fifty-five children met the inclusion criteria and participated in the 1-year study. Decay, missing, filling teeth (Dmft) was in the intermediate category (4.13) at baseline and fell into the high category (5.16) at 1-year follow-up, although the increase in caries remained in the low category.

**Conclusion** Dysplastic children with a history of neonatal developmental delay experience dental caries beginning in the first year of life and may become more severe later in life.

## Introduction

Stunting occurs in 155 million children worldwide and is an indicator of a child's overall well-being and can indicate social inequalities. 1 Stunting indicates chronic limitation of a child's growth potential due to insufficient nutrient intake. 2 The diagnosis of stunting refers to height for age which has been set by the Child Growth Standards Median, World Health Organization (WHO). 3 4 The national stunting prevalence target suggested by WHO should be below 20%, but in 2019, Indonesia still has a fairly high national stunting prevalence which is 27.67%. 3 4 5

Stunting that starts intrauterine will increase the likelihood of a baby being born with a stunted state, thus showing the presence of malnutrition that is passed down between generations. 6 The causes of stunting, especially in Indonesia, include the maternal knowledge and level of education, baby's birth length, exclusive breastfeeding for 6 months, the incidence of infection, and the family's socioeconomic status. 4 Other risk factors that can increase the incidence of stunting are the child's birth weight less than 2,500g and the mother's body posture, including height <145cm and weight <40kg before pregnancy. Neonatal stunting can be seen from the baby's body length at birth which is less than 48cm and in the first 30 days after birth, the baby does not experience significant changes in body weight. 6 7 Children who are stunted at birth will experience a permanent impact on the physical, cognitive, health, and economic spheres in the future, so that neonatal stunting is an indicator of birth outcomes from the intrauterine environment and as a predictor of child growth and development. 8 The nutritional, socioeconomic, and environmental conditions of children growing will not be much different from the conditions that cause children to experience neonatal stunting, so early intervention on stunting is needed to suppress the adverse effects on prolonged child development. 9

According to Indonesian health research data in 2018, dental caries in children aged 3 to 4 and 5 to 9 years reached the prevalence of 81.5 and 92.6%, respectively. 10 Malnutrition that occurs in children will affect the health condition of the oral cavity, especially the teeth. 11 Stunting children are at high risk for caries because there is atrophy of the salivary glands which can cause a decrease in salivary buffering, self-cleansing, antisolvent, and antibacterial functions saliva. 11 12 13 The salivary flow rate will also decrease in chronically malnourished children, whether stimulated or not, to increase the cariogenic potential of the teeth. 14 Nutritional disorders in children with low birth weight (LBW) can cause cloudiness on the tooth surface and enamel hypoplasia, one of the predisposing factors for the Early Childhood Caries (ECC). 15 Therefore, dental caries is one aspect that needs to be considered in children who experience stunting. The incidence of stunting in Bandung in 2019 was recorded as 8.121 of the 124.319 children (6.64%) under 5 years. District Sukajadi, as part of Bandung city, has 183 stunting cases in infants aged 0 to 59 months. 16 This study aimed to analyze the dental caries experience in stunting children with also a history of neonatal stunting.

## Materials and Methods

### Study Design

A descriptive, analytic study used the population of children who experienced growth stunting, aged 0 to 59 months, recorded at two health centers in Sukajadi District, Bandung, West Java, as many as in 164 children. Sampling in this study using a convenient sampling technique. Stunting children, who had a birth length below the normal value (<48cm), aged under 48 months (for first-year follow-up), have a record in his health book that does not experience significant weight gain in the first 30 days after birth, and has experienced the eruption of his first deciduous teeth, were included in this study. Ethical review and approval were approved by the Research Ethics Committee of Universitas Padjadjaran, file no. 676/UN6.KRP/EC/2020 and expanded by 094/UN6.KEP/EC/2021.

### Data Collection

The current nutritional status of children is assessed by measuring their height and weight. Trained cadres use distance meters and electronic scales to measure and weigh children wearing loose-fitting shoes and light clothing. The history of the child's neonatal status from birth was obtained from the child control book to the integrated health post. These cadres also interview the mothers in a setting that is as private as possible. The interview questions consist of oral hygiene practice, dental care experience, and history of a child's diet during infancy.

A licensed dentist can only perform a dental examination on a child by performing a visual inspection using an oral mirror and a light. Caries, missing, and filled teeth (decay missing filling=dmf); cavitation depth and examination; deep cavitation only in enamel, dentin, and near or in pulp were documented. In addition, examiners briefly standardized their tests by examining five children independently and then together and agreeing on the results.

### Data Analysis

Data were recorded on paper forms and manually entered into a Microsoft Excel spreadsheet for later analysis using IBM SPSS (version 23.0). After assessment, descriptive statistics was generated prior to bivariate analysis. A child's experience with dental caries was analyzed in terms of the presence of decay and the degree or severity of caries, calculated as (1) the mean number of caries, missing, or filling teeth (dmft); (2) the presence of a deep cavity; (3) “carious increment” is defined as the number of new caries per year which is divided into low caries (0–2 dmf/year), (3–5 dmf/year), and high (≥6 dmf/year) year); and (4) the presence of mouth pain reported by the mother.

## Result

### Patient Characteristics

A total of 164 stunted children aged 0 to 59 months participated in a population-based sample. This number included 114 patients aged 0 to 48 months in the base year (2020) and suitable for 1-year follow-up (<6 years in 2021). Of these 114 patients, 55 met the inclusion criteria and had a birth history <48cm. The children were aged 29 to 58 months (mean=44 months, standard deviation=8.59), 30 boys and 25 girls.

### Nutrition and Oral Health Practices

Almost all patients received breast milk, only 20% were also bottle-fed. Children regularly visit integrated-health postactivities held in their village and are always advised on balanced nutrition. For oral health practices, 71% of mothers brush their children's teeth, although only 33% regularly brush their teeth and 13% visit the dentist ( Table 1 ).

**Table 1:** Patient characteristics and nutrition/oral health practice

	Baseline (2020)
*n* =55	
Child mean age (mo)	Mean=44, SD=8.59
Child gender (%)	Male=30, female=25
Nutrition practices (%)	
Child ever breast fed	71
Child ever bottle fed	29
Oral health practice (%)	
Mother brushes child's teeth	71
Routinely brush teeth	33
Child ever been to the dentist	13

Abbreviation: SD, standard deviation.

Table 2 shows the nutritional status of the patients at baseline and 1-year follow-up. The examination was performed by comparing the child's height with the child's age and then analyzing the nutritional status based on the *z* -score criteria set by WHO. The percentages between patients born with moderate and low weight were almost equal. The data below show that children who experience neonatal stunting have a short nutritional status, as seen from the height per age of 81.8%. Meanwhile, when viewed from the weight per height is still in the category of good nutrition (80%).

**Table 2:** Characteristics of patients based on nutritional status

Nutritional status (height per age)	2020		2021	
	*n*	%	*n*	%
Low birth weight	29	52.7		
Adequate birth weight	26	47.3		
Short stature	45	81.8	44	80
Very short stature	10	18.2	11	20
Good nutrition	44	80	44	80
Malnutrition	5	9	5	9
Malnutrition	1	1.8	1	1.8
More nutrition	1	1.8	1	1.8
More nutritional risk	4	7.4	4	7.4

### Early Childhood Caries and Oral Pain

**Fig. 1:**
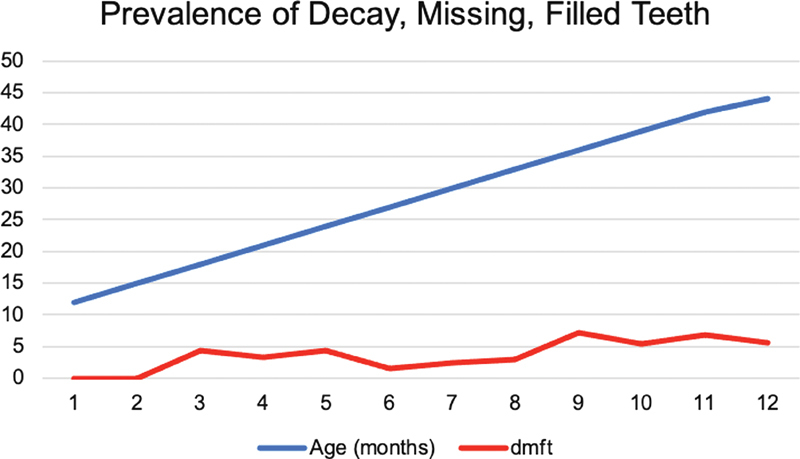
Prevalence of decay, missing, and filling teeth per age. Dmft, decay, missing, filling teeth.

Dental caries in many patients started in the first year of life ( Fig. 1 ). Table 3 presents information on the total number of primary teeth in children with cavities (d-t), teeth that have been filled (f-t), and teeth that have been lost or extracted due to caries (m-t). The group dmf-t index value is moderate (4.13) and high (5.16) in 2020 and 2021, respectively, according to the WHO index category. The prevalence of deep cavitation increased but oral pain decreased.

**Table 3:** Components of d-t, f-t, m-t in children with neonatal stunting

	2020	2021
Dmft	4.13	5.16
Prevalence of deep cavitation	14.5%	20%
Prevalence of oral pain	12.7%	7.27%
Caries increment		1.07

Abbreviations: d-t, total number of primary teeth in children with cavities;Dmft, decay, missing, filling teeth; f-t, teeth that have been filled; m-t, teeth that have been lost or extracted due to caries.

## Discussion

This study has a sample of 55 children with a history of neonatal stunting where the percentage of males is higher than that of females ( Table 4 ). This study follows the study by Akombi et al, noted that boys are more likely to be affected by stunting than girls. 17 Research by Gonete et al also shows that it is 2.9 times more likely than boys are born with stunting conditions than girls. 8 Jahanihashemi et al said that according to his research, the association of stunting with gender was not very significant, but a factor that was very closely related was the incidence of stunting with the characteristics of the environment where the child lived. 18

**Table 4:** Caries rates in children with neonatal stunting

dmft	*n*	%
Very low	11	20
Low	13	23.6
Moderate	9	16.4
High	7	12.7
Very high	15	27.3
	55	100

Abreviation: Dmft, decay, missing, filling teeth.

All children were born with a length of less than 48cm, indicating that the children were born underdeveloped. According to research conducted by Schmidt et al, the important determinants in reflecting the nutritional status of children in Indonesia are length and weight at birth. 19 20 Therefore, the child's birth length, which is a predictor of nutritional status, should be optimized in the womb by preparing adequate maternal nutritional status. According to research by Akombi et al, maternal body mass index (BMI) less than 18.5, children who have experienced diarrhea, and children living in families below the poverty line have a higher likelihood of experiencing stunting. 17 Another study also explained that mothers with short conditions increased 2.8 times their children were born stunted, and mothers who experienced chronic malnutrition caused 15.4 times their children were born with stunting conditions. 8

The study also described the child's birth weight is one of the risk factors for stunting in newborns. Also, 52.7% of the children had a birth weight of 2,500g, indicating that the children belonged to the LBW category. The child's weight at birth will determine the child's general growth in the future, so that children with LBW will experience less growth in both height and weight. 18 Consistent with the study, Gonete et al noted that LBW was associated with a 3.2-fold increase in the incidence of neonatal stunting compared with infants of reasonable weight. 8 The study by Titaley et al also showed that in Indonesia, children with birth weight <2,500g were 2.55 times more likely to be stunted. 21 LBW are six variables used by Aryastami et al. The variables studied were most strongly associated with stunting. 7

The children in the study were stunned when they investigated. A total of 45 infants with neonatal developmental delay were short and 10 were very short. Children who are delayed are twice as likely to be stunted at 2 years of age. 8 Other studies also explain that the vulnerable stage of erratic growth is in the womb and the first 2 years of the child's life. 22 Stunting in children is detrimental because most children with short stature get sunburned in adulthood. 9 Children born with short stature can still achieve a certain height, but they do not reach the same height as children born with normal height. 20 Therefore, a child's birth length plays an important role in determining a child's future height growth.

The data study showed that the dmf-t index of the neonatal stunting group was included in the high group. This figure clearly shows that every child has at least six cavities, fillings, or cavities. This study is consistent with the study by Abdat who demonstrated a strong association between stunting and children's oral and dental health. A study in Aceh, Indonesia, showed that children with stunting had a def-t index of 6.13 compared with 3.7 for normal children. 23

Longitudinal study by Dimaisip-Nabuab et al, studies in Cambodia, Indonesia, and Laos also show an association between primary caries and chronically malnourished children. 24 More than one-third of children had a very high experience of dental caries, and as many as 13 children had a high and moderate experience of dental caries. The study by Vieira et al supports the high caries rates in malnourished children which explains that one of the factors that may be low salivary flow rate that can exacerbate carious conditions in primary teeth. 25 Vitamin-A deficiency may be a factor associated with salivary gland atrophy. 26 According to the study by Abdat, atrophy of salivary glands in children with developmental delay may reduce self-cleaning process, useful for oral health protection. 23 Malnutrition, which indicates a lack of micronutrients, such as vitamins, iron and zinc, reduces the protective properties of saliva to prevent oral infections. Based on a review of Sheetal et al, malnutrition contributes to the risk of dental caries in many affected children. 26

The study included 29 LBW children. Masumo et al said in their study that LBW children had more plaque on the tooth surface. 15 Nelson et al also found that children with very LBW were more likely to develop enamel hypoplasia. 27 Enamel hypoplasia that occurs will increase the likelihood of children having ECC. 28 Thus, LBW is a factor in childhood stunting and is associated with a high caries index in malnourished children.

Child malnutrition must be a major problem because according to WHO, malnutrition accounts for around 45% of deaths in children under 5 years. Prevention of stunting in children must be adequate and timely. Growth retardation can be prevented between 1,000 days after fertilization and 2 years of age by maintaining prenatal and postnatal nutritional intake. Stunting requires sustained intervention across different sectors, so that its impact can improve the quality of life in communities and even boost national economies in addition to reducing the burden of disease. A combination of interventions ranging from improved nutrition and infection control (including water quality, sanitation, and hygiene) to a comprehensive focus on maternal and child health before and after pregnancy (antenatal care) can prevent child stunting. 29

According to Khoeroh and Indriyanti, pregnancy screening (antenatal care [ANC]) for pregnant women is a form of prevention that can be used to detect the various risks that may arise early; mothers with an ANC less than four times as many had a 2.4 times higher risk of having a child with stunting. 30 Gaining less than 9.1kg increases the chances of a child being born smaller. 31 Therefore, maternal weight gain during pregnancy must also be considered as a benchmark for optimal nutritional intake. 2 The psychological state of the mother during pregnancy is also important because according to the study by Wemakor and Mensah. Depressed mothers may increase the likelihood of a child being born with stunted growth compared with mentally healthy mothers. 32

Prevention of developmental delay and prevention of cavities in children can also be coordinated through improved dental and oral health education, brushing habits, the use of fluoride, and the introduction of dental care from an early age. 33 The role of the dentist in the oral health of children with developmental delays can also be curative. For example, severe tooth decay in a child with developmental delay should be treated to restore the function of the tooth and improve the child's oral health. A limitation of this study is the lack of complementary data on risk factors for growth stunting, such as height, weight, maternal BMI, frequency of infection, breastfeeding, maternal education, and other external factors.

## Conclusion

In conclusion, this local survey found that the dental caries experience in hypoplastic children with a history of neonatal developmental delay begins in the first year of life and may become more severe later in life. But sociodemographic characteristics, dental health behaviors, and dietary habits are also effective in maintaining oral and dental health. Therefore, dental and oral health support measures should be linked to dental health education programs, especially for those facing the dual burden of diet and dental and oral health. Also, maintaining oral hygiene practices can be very important for dental health policy makers.

## References

[1] de Onis M Borghi E Arimond M Prevalence thresholds for wasting, overweight and stunting in children under 5 years Public Health Nutr 2019 22 01 175 179 30296964 10.1017/S1368980018002434 PMC6390397

[2] Chirande L Charwe D Mbwana H Determinants of stunting and severe stunting among under-fives in Tanzania: evidence from the 2010 cross-sectional household survey BMC Pediatr 2015 15 01 165 26489405 10.1186/s12887-015-0482-9 PMC4618754

[3] Bouma S Diagnosing pediatric malnutrition: paradigm shifts of etiology-related definitions and appraisal of the indicators Nutr Clin Pract 2017 32 01 52 67 10.1177/0884533616671861 30865345

[4] Beal T Tumilowicz A Sutrisna A Izwardy D Neufeld L M A review of child stunting determinants in Indonesia Matern Child Nutr 2018 14 04 e12617 29770565 10.1111/mcn.12617 PMC6175423

[5] Indriyanti R Nainggolan T R Sundari A S Chemiawan E Gartika M Setiawan A S Modelling the maternal oral health knowledge, age group, social-economic status, and oral health-related quality of life in stunting children Int J Stat Med Res 2021 10 01 200 207

[6] Sumarmi S Maternal short stature and neonatal stunting: an inter-generational cycle of malnutritionPresented at: International Conference on Health and Well-Being: Surakarta, Indonesia; 2016:265–272

[7] Aryastami N K Shankar A Kusumawardani N Besral B Jahari A B Achadi E Low birth weight was the most dominant predictor associated with stunting among children aged 12–23 months in Indonesia BMC Nutr 2017 3 01 16

[8] Gonete A T Kassahun B Mekonnen E G Takele W W Stunting at birth and associated factors among newborns delivered at the University of Gondar Comprehensive Specialized Referral Hospital PLoS One 2021 16 01 e0245528 33471862 10.1371/journal.pone.0245528 PMC7817059

[9] Dewey K G Begum K Long-term consequences of stunting in early life Matern Child Nutr 2011 7 03 5 18 21929633 10.1111/j.1740-8709.2011.00349.x PMC6860846

[10] Dinas kesehatan. Riskesdas 2018 Jakarta: Badan Penelitian dan Pengembangan Kesehatan Departemen Kesehatan Republik Indonesia2018. 93–94 p.

[11] Abdat M Usman S Chairunas C Suhaila H Relationship between stunting with dental and oral status in toddlers Journal of Dentomaxillofacial Science. 2020 5 02 114 119

[12] Setiawan A S Abhista N Andisetyanto P Indriyanti R Suryanti N Growth stunting implication in children: a review on primary tooth eruption Eur J Gen Dent 2022 11 01 7 16

[13] Sadida Z J Indriyanti R Setiawan A S Does growth stunting correlate with oral health in children?: a systematic review Eur J Dent 2022 16 01 32 40 34598296 10.1055/s-0041-1731887 PMC8890921

[14] Kaur S Soni S Prashar A Bansal N Brar J S Kaur M Clinical and radiographic evaluation of autogenous dentin graft and demineralized freeze-dried bone allograft with chorion membrane in the treatment of grade ii and iii furcation defects-: a randomized controlled trial Indian Journal of Dental Sciences. 2019 11 02 10 13

[15] Masumo R Bårdsen A Astrøm A N Developmental defects of enamel in primary teeth and association with early life course events: a study of 6-36 month old children in Manyara, Tanzania BMC Oral Health 2013 13 01 21 23672512 10.1186/1472-6831-13-21 PMC3671208

[16] Dinas Kehesatan Kota Bandung. Profil Kesehatan Kota Bandung TahunAvailable at: Profil-Kesehatan-Kota-Bandung-Tahun-2019.pdf. Accessed May 18, 2022

[17] Akombi B J Agho K E Hall J J Merom D Astell-Burt T Renzaho A MN Stunting and severe stunting among children under-5 years in Nigeria: a multilevel analysis BMC Pediatr 2017 17 01 15 28086835 10.1186/s12887-016-0770-z PMC5237247

[18] Jahanihashemi H Noroozi M Zavoshy R Afkhamrezaei A Jalilolghadr S Esmailzadehha N Malnutrition and birth related determinants among children in Qazvin, Iran Eur J Public Health 2017 27 03 559 562 28472277 10.1093/eurpub/ckx043

[19] Schmidt M K Muslimatun S West C E Schultink W Gross R Hautvast J GAJ Nutritional status and linear growth of Indonesian infants in west java are determined more by prenatal environment than by postnatal factors J Nutr 2002 132 08 2202 2207 12163663 10.1093/jn/132.8.2202

[20] GBD 2017 Disease and Injury Incidence and Prevalence Collaborators James S L Abate D Abate K H Global, regional, and national incidence, prevalence, and years lived with disability for 354 diseases and injuries for 195 countries and territories, 1990-2017: a systematic analysis for the Global Burden of Disease Study 2017 Lancet 2018 392(10159):1789 1858 30496104 10.1016/S0140-6736(18)32279-7 PMC6227754

[21] Titaley C R Ariawan I Hapsari D Muasyaroh A Dibley M J Determinants of the stunting of children under two years old in Indonesia: a multilevel analysis of the 2013 Indonesia basic health survey Nutrients 2019 11 05 13 10.3390/nu11051106 PMC6567198 31109058

[22] de Onis M Blössner M Borghi E Prevalence and trends of stunting among pre-school children, 1990-2020 Public Health Nutr 2012 15 01 142 148 21752311 10.1017/S1368980011001315

[23] Abdat M Stunting pada balita dipengaruhi kesehatan gigi geliginya Journal Of Syiah Kuala Dentistry Society 2019 4 02 36 40

[24] Dimaisip-Nabuab J Duijster D Benzian H Nutritional status, dental caries and tooth eruption in children: a longitudinal study in Cambodia, Indonesia and Lao PDR BMC Pediatr 2018 18 01 300 30217185 10.1186/s12887-018-1277-6 PMC6137874

[25] Vieira K A Rosa-Júnior L S Souza M AV Santos N B Florêncio T MMT Bussadori S K Chronic malnutrition and oral health status in children aged 1 to 5 years: an observational study Medicine (Baltimore) 2020 99 18 e19595 32358344 10.1097/MD.0000000000019595 PMC7440136

[26] Sheetal A Hiremath V K Patil A G Sajjansetty S Kumar S R Malnutrition and its oral outcome - a review J Clin Diagn Res 2013 7 01 178 180 23449967 10.7860/JCDR/2012/5104.2702 PMC3576783

[27] Nelson S Albert J M Geng C Increased enamel hypoplasia and very low birthweight infants J Dent Res 2013 92 09 788 794 23857641 10.1177/0022034513497751 PMC3744269

[28] Folayan M O El Tantawi M Oginni A B Alade M Adeniyi A Finlayson T L Malnutrition, enamel defects, and early childhood caries in preschool children in a sub-urban Nigeria population PLoS One 2020 15 07 e0232998 32609719 10.1371/journal.pone.0232998 PMC7329100

[29] de Onis M Blössner M The World Health Organization Global Database on Child Growth and Malnutrition: methodology and applications Int J Epidemiol 2003 32 04 518 526 12913022 10.1093/ije/dyg099

[30] Khoeroh H Indriyanti D Evaluasi penatalaksanaan gizi balita stunting di wilayah kerja Puskesmas Sirampog Unnes J Public Heal 2017 6 03 189 195

[31] Trihono A Tjandrarini D H Irawati A Utami N H Tejayanti T Pendek (Stunting) di Indonesia, Masalah dan Solusinya. Lembaga Penerbit Balitbangkes Jakarta Lembaga Penerbit Balitbangkes 2015:245

[32] Wemakor A Mensah K A Association between maternal depression and child stunting in Northern Ghana: a cross-sectional study BMC Public Health 2016 16 01 869 27557725 10.1186/s12889-016-3558-z PMC4997709

[33] Sokal-Gutierrez K Turton B Husby H Paz C L Early childhood caries and malnutrition: baseline and two-year follow-up results of a community-based prevention intervention in Rural Ecuador BMC Nutr 2016 2 01 1 11

